# Location of tumor affects local and distant immune cell type and number

**DOI:** 10.1002/iid3.144

**Published:** 2017-02-23

**Authors:** Jonathan A. Hensel, Vinayak Khattar, Reading Ashton, Carnellia Lee, Gene P. Siegal, Selvarangan Ponnazhagan

**Affiliations:** ^1^ Department of PathologyUniversity of Alabama at BirminghamBirminghamAlabamaUSA

**Keywords:** Dendritic cells, macrophages, tumor location, T‐cells

## Abstract

**Introduction:**

Tumors comprise heterogeneous populations of cells, including immune infiltrates that polarize during growth and metastasis. Our preclinical studies on breast cancer (BCa) identified functional differences in myeloid‐derived suppressor cells based on tumor microenvironment (TME), prompting variations in host immune response to tumor growth, and dissemination based on tissue type.

**Methods:**

In order to understand if such variations existed among other immune cells, and if such alteration occurs in response to tumor growth at the primary site or due to bone dissemination, we characterized immune cells, examining localized growth and in the tibia. In addition, immune cells from the spleen were examined from animals of both tumor locations by flow cytometry.

**Results:**

The study demonstrates that location of tumor, and not simply the tumor itself, has a definitive role in regulating immune effectors. Among all immune cells characterized, macrophages were decreased and myeloid dendritic cell were increased in both tumor locations. This difference was more evident in subcutaneous tumors. Additionally, spleens from mice with subcutaneous tumors contained greater increases in both macrophages and myeloid dendritic cells than in mice with bone tumors. Furthermore, in subcutaneous tumors there was an increase in CD4^+^ and CD8^+^ T‐cell numbers, which was also observed in their spleens.

**Conclusions:**

These data indicate that alterations in tumor‐reactive immune cells are more pronounced at the primary site, and exert a similar change at the major secondary lymphoid organ than in the bone TME. These findings could provide translational insight into designing therapeutic strategies that account for location of metastatic foci.

## Introduction

It is well established that that the tumor microenvironment plays an important role in regulating the growth of primary tumors and during metastasis 1, 2, 3. Immune infiltrates represent a major component the tumor microenvironment (TME) 4, 5, 6, 7. The mass of some breast tumors have been demonstrated to consist of as much as 50% macrophages 8. These infiltrating immune effector cells can be conscripted by cancer cells and aberrant stromal tissue into promoting tumor survival and growth 9, 10, 11, 12. Tumor, stroma, and immune cells form networks of crosstalk, which in‐turn induces production of a milieu of protumorigenic cytokines leading to a suppression of the immune system that promotes further local tumor growth and metastasis 13, 14, 15, 16, 17. Bone is a common metastatic site of breast cancer (BCa) 18. When BCa cells colonize bone, they trigger osteolytic damage, which permits tumor expansion. The canonical model describing this process entails secretion of tumor‐derived factors, especially receptor activator of nuclear factor kappa‐B ligand), which stimulates activation of osteoclast precursors to mature osteoclast, which then resorb bone, leading to the release of protumorigenic growth factors and formation of a niche conducive of tumor growth 19, 20, 21. Previously, we and others have shown that myeloid‐derived suppressor cells (MDSC) function as a direct source of osteoclasts 22, 23, 24. An important finding in our studies was only MDSC within the bone TME differentiated into osteoclast, whereas MDSC from non‐bone sites of BCa metastasis or from spleen did not 22. This led us to hypothesize that metastatic BCa tumor location itself can play an important role on the type, number and polarization of immune cells within the TME. Additionally, we hypothesize metastatic BCa tumor location dictates immune profiles at immunologically important sites outside the TME. To test this, we utilized a syngeneic murine BCa cell line in an immunocompetent mouse model and characterized immune cells in response to tumor location. To determine if tumor site exerts influence on immune cells systemically, we examined the spleen, an important lymphoid organ for priming immune effectors. Results of this study indicated that location of tumor, and not simply the tumor itself, exerts a definitive role in regulating immune effector populations. Significant alterations were noted among macrophages, myeloid dendritic cells (mDC), and CD4^+^ and CD8^+^ T‐cells. These findings could provide translational insight into designing therapeutic strategies that account for location of primary tumor and metastatic foci.

## Materials and Methods

### Cell culture

The murine breast cancer cell line 4T1 fLuc, constitutively expressing firefly luciferase were kind gifts from Dr. Xiaoyuan Chen (Stanford University, Stanford, CA) 22, 25. Cells were maintained in a cell culture incubator at 37°C with 5% CO_2_ and cultured in RPMI 1640 media, 10% FBS, 10 mM HEPES, 1 mM Na‐Pyruvate, 4.5 mg/L Glucose, 1% Pen‐Strep, and 100 μg/ml of G418.

### In vivo tumor models

For tumor implantation experiments within the bone, 6–8‐week‐old female BALB/c mice were intratibially injected with 5 × 10^4^ 4T1 fLuc cells. Control, sham injections were made with PBS. Tumor engraftment was monitored by non‐invasive luciferase imaging and mice were sacrificed 7 days post injection and immune cells were collected from bone marrow and spleen for flow cytometry. For subcutaneous tumor implantation experiments, 6–8‐week‐old female BALB/c mice were injected with 2 × 10^5^ syngeneic 4T1fLuc cells. When palpable tumors of approximately 100 mm^3^ had developed, mice were sacrificed and bone marrow, tumor and splenic cells were collected. Animal care and treatments were conducted in accordance with established guidelines and protocols approved by the UAB Institutional Animal Care and Use Committee.

### Luciferase image analysis

Progression of the 4T1 tumor growth was followed by non‐invasive imaging of mice using the IVIS Imaging System (Xenogen Corp., Alameda, CA). Briefly, mice were anesthetized using isoflurane gas and intraperitoneally injected with d‐luciferin (150 mg/kg body weight) and were placed in a light‐tight chamber. The photographic (gray‐scale) reference image was obtained at 10 min after d‐luciferin injection, and the bioluminescent image was collected immediately thereafter. Data acquisition software was calibrated so that no pixels were saturated during image collection. The bioluminescent and gray‐scale images were overlaid using Living Image Software (Xenogen). Living Image Software was also used to obtain an image representing bioluminescence intensity, with blue being the least intense, and red the most intense). Intratibial injected mice were imaged on day 0 for bioluminescent signal after injection of 4T1 fLuc cells for baseline readings of tumor burden. Imaging was performed again on days 3 and 6 before sacrifice on day 7. Localized subcutaneous 4T1 fLuc tumor engraftment was also established with luciferase imaging.

### Isolation of immune cells and flow cytometry

At time of sacrifice, cells were collected for flow cytometry. Dissociation of tumor tissue was performed using Miltenyi Biotec Tumor Dissociation Kit. Tumors were minced with scalpel and incubated in dissociation solution at 37°C on shaker for a minimum of 30 min followed by passage through a 100 μm sterile cell strainer (Thermo Fisher Scientific, Waltham, MA). Bone marrow cells were acquired via flushing of bone marrow cavities with FACS buffer (PBS with 3% FBS) using insulin syringe with 28G needle, followed with passage through a 100 μm sterile cell strainer. Splenic cells were acquired using FACS buffer and gentle pressure‐dissociation of spleen and passed through a 100 μm sterile cell strainer. For all experiments, single cell suspensions were washed with FACS buffer and pelleted. Red blood cells in pellet were lyzed by addition of 5 ml RBC lysis buffer (BioLegend, San Diego, CA), vortexed briefly to suspend cells and incubated for 5 min, followed by FACS buffer wash and spin. Cells were resuspended in FACS buffer and incubated with Fc block at 4°C for 15 min. Cells were divided into individual tubes for respective cell type analysis, suspended in 100 µl of FACS buffer and stained for the following cell types with all antibodies for 30 min at 4°C. All antibodies were purchased from eBioscience, (San Diego, CA) unless noted otherwise. Macrophages were stained with one of the following combinations to assess phenotypes; (1) CD11b eFluor450 (cat# 48‐0112) + F4/80 Allophycocyanin (APC) (cat# 17‐4801) + CD80 Fluorescein (FITC) (cat# 11‐0801) + CD86 Phycoerythrin (PE) (cat# 12‐0861); (2) CD11b eFluor450 (cat# 48‐0112) + F4/80 APC (cat# 17‐4801) + MHCII APC‐eFluor780 (cat# 47‐5321) + iNOS PE (cat# 12‐5920); (3) CD11b eFluor450 (cat# 48‐0112) + F4/80 PE‐Cy7 (cat# 25‐4801) + CD206 APC (R&D Systems, Minneapolis, MN, cat# FAB2535A) + Arginase FITC (R&D Systems, cat# IC5868F). After surface staining, combinations 2 and 3 were then fixed and permeabilized according to manufacturer's protocol prior to staining for iNOS and Arginase antibodies, respectively. Myeloid dendritic cells (mDC) were stained with CD11b APC (cat# 17‐0112), CD11c APC‐eFluor780 (cat# 47‐0114), and MHCII FITC (cat# 11‐5321). CD4^+^ and CD8^+^ T‐cells were stained with CD3 APC‐eFluor780 (cat# 47‐0032), CD4 eFluor450 (cat# 48‐0041), and CD8 APC (cat# 17‐0081). T‐regs were stained with CD3 APC‐eFluor780 (cat# 47‐0032), CD4 eFluor450 (cat# 48‐0041), CD25 PE (cat# 12‐0251), and FoxP3 APC (cat# 17‐5773). After surface staining, T‐regs were fixed and permeabilized according to manufacturer's protocol prior to staining for intracellular FoxP3. Natural killer cells (NK) were stained with CD69 FITC (cat# 11‐0691) and CD49b PE (cat# 12‐5971). Plasmacytoid dendritic cells (pDC) were stained with CD11c APC‐eFluor780 (cat# 47‐0114), B220 PerCp‐Cy5.5 (cat# 45‐0452), and Siglec‐H PE (cat# 12‐0333). After washing with PBS, cells were analyzed using a BD FACS LSRII (BD Biosciences, San Jose, CA) followed with analysis using FlowJo software program (FlowJo, Ashland, OR).

### Statistical analysis

Data were analyzed by Student's *t* test. Values provided are the Mean ± SEM and the differences were considered significant if *p *< 0.05. Sample values greater than ±1.7 STD were excluded from analysis.

## Results

### Establishment of BCa tumors in bone and subcutaneous locations

**Figure 1:**
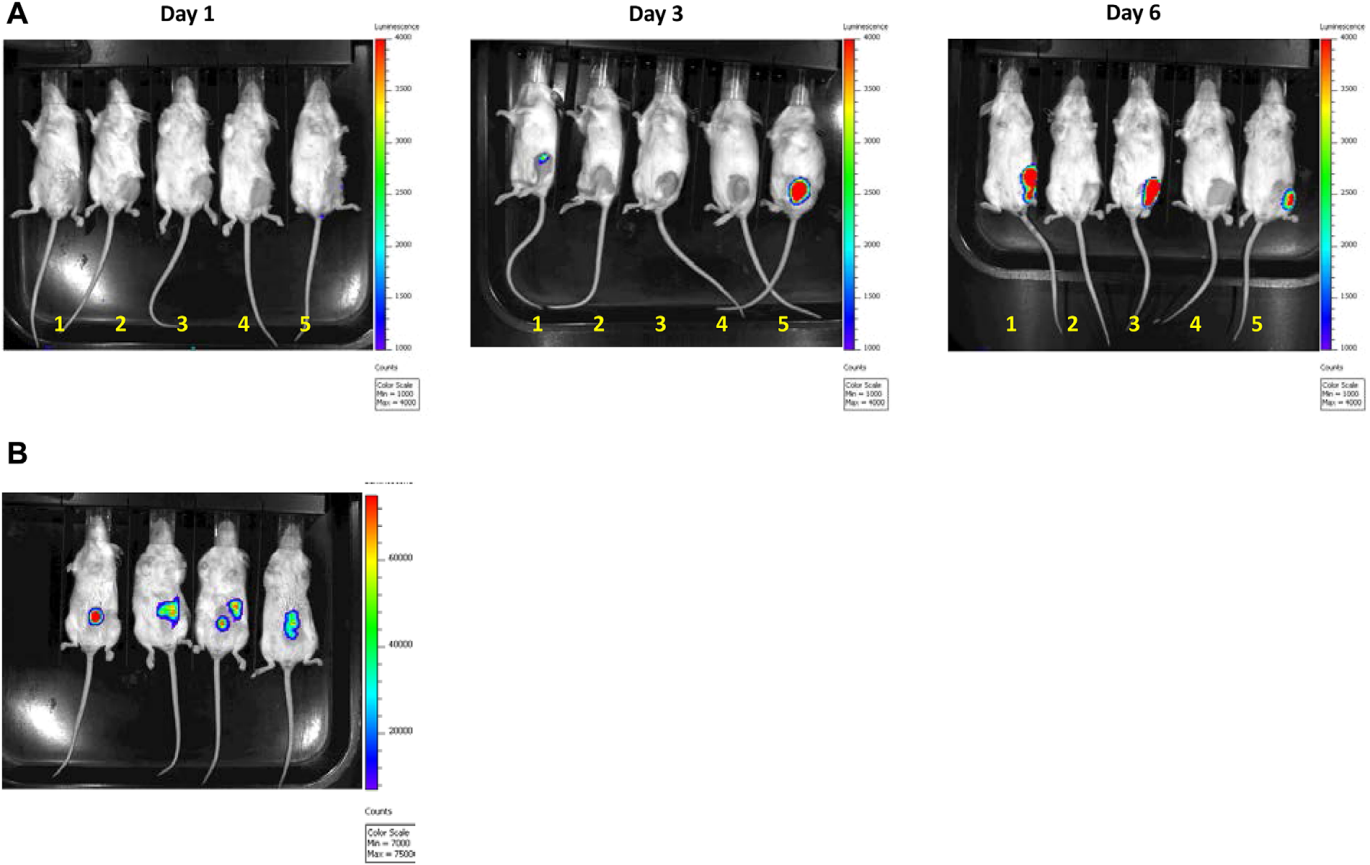
Non‐invasive imaging of tumor growth in vivo. Post injection luciferase imaging. Luciferase imaging on days 1, 3, and 6 confirmed tumor establishment and increasing tumor burden in the injected tibias of mice with no dissemination to other sites. Balb/C mice in positions 1, 3, and 5 were injected with 5 × 10^4^ syngeneic breast cancer 4T1 cells expressing luciferase (4T1 fLuc) in left tibia. Mice in position 2 and 4 were PBS sham injection controls (A). Luciferase imaging confirmed localized tumors in subcutaneous region, injected with 2.5 × 10^4^ 4T1fLuc cells (B).

In order to exclusively determine variations in immune infiltrates during tumor growth within subcutaneous site and bone microenvironment, tumor cell transplantation was selectively localized. We utilized the breast cancer cell line 4T1 fLuc, constitutively expressing firefly luciferase, syngeneic to BALB/c mice. The use of 4T1 fLuc cells enabled non‐invasive assessment of tumor establishment. Luciferase imaging for intratibial injections was performed at multiple time points to establish tumor take and increasing tumor burden. Increasing luciferase signal at successive time points demonstrated presence and continued tumor growth in 4T1 fLuc injected mice versus no signal for PBS sham injected mice and furthermore that tumor growth was restricted to a singular site within the injected tibia (Fig. 1A). Localized subcutaneous 4T1 fLuc tumor engraftment was also observed with luciferase imaging (Fig. 1B).

### Subcutaneous tumor location has greater effect on macrophage infiltration than intratibial location

**Figure 2:**
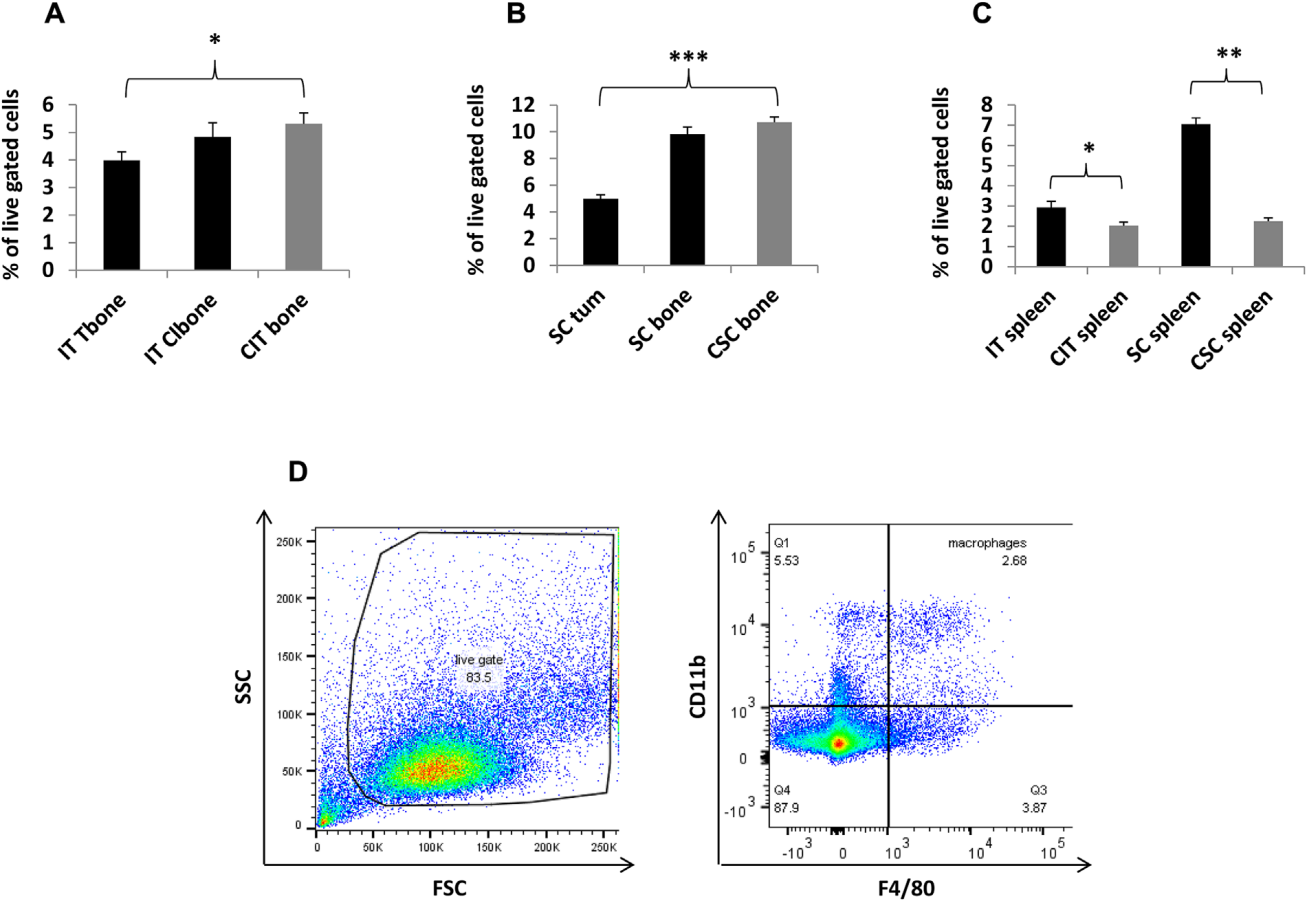
Comparative flow cytometry analysis of macrophages in bone, spleen, and tumors: Macrophages were decreased within the bone and subcutaneous TME's compared to controls (A and B, respectively). The difference was more pronounced in subcutaneous tumors than intratibial tumors (A and B). Macrophages in spleens of tumor bearing mice were increased in comparison to controls (C). This increase was more pronounced in subcutaneous tumor versus intratibial tumor bearing mice, compared to controls (C). Representative flow cytometry gating for macrophages is shown (D). Tissue sites examined were intratibial tumor injected bone (IT Tbone), intratibial contralateral bone (IT Clbone), PBS intratibial injected bone (CIT bone), spleen from intratibial tumor injected animals (IT spleen), spleen from intratibial PBS injected animals (CIT spleen), subcutaneous tumor (SC Tum), non‐tumor bearing bone from subcutaneous tumor injected mice (SC bone), bone from subcutaneous control mice (CSC bone), spleen from subcutaneous injected mice (SC spleen), spleen from subcutaneous control mice (CSC spleen) IT injected tumor group; *n* = 9, IT control group; *n* = 6, subcutaneous tumor group; *n* = 7, subcutaneous control group; *n* = 3. (*) denotes *p* < 0.05, (**) denotes *p* < 0.01, (***) denotes *p* < 0.001.

To test the hypothesis that tumor location exerts a role in regulating tumor and systemic resident immune effector cells, we injected 4T1 fLuc tumor cells intratibially and subcutaneously, then used FACS analysis to determine effects on immune cell populations. To narrow our focus, we conducted preliminary experiments that examined multiple cell types including dendritic cells, both myeloid and pDC, macrophages, NK, neutrophils, and T‐cells. Among these, pDC, NK, and neutrophils did not have significant differences between the two tumor sites and were eliminated from further study (Fig. S1). However, significant differences did occur among macrophages. While macrophages were decreased within the bone and subcutaneous TME's compared to respective controls (Fig. 2A and B, respectively), the effect was more pronounced in subcutaneous tumors with a 59% decrease (Fig. 2B) compared to a 27% decrease within intratibial tumors (Fig. 2A). In contrast to this decrease within the TME, there was an increase in splenic macrophages of tumor‐bearing mice compared to controls. This increase was more pronounced in mice bearing only subcutaneous tumors, which had a 267% increase compared to a 53% increase within intratibial tumors (Fig. 2C). Representative flow cytometry gating for macrophages is shown (Fig. 2D). When examining if tumor location affects M1 versus M2 phenotype, we saw no significant difference. Ratios between tissue sites for both tumor locations remained constant. Levels of both phenotypes merely followed levels of total macrophages; implying tumor location does not alter macrophage phenotype at this time point in tumor development (Fig. S2).

### Subcutaneous tumor's effect on mDC extend beyond the TME

**Figure 3:**
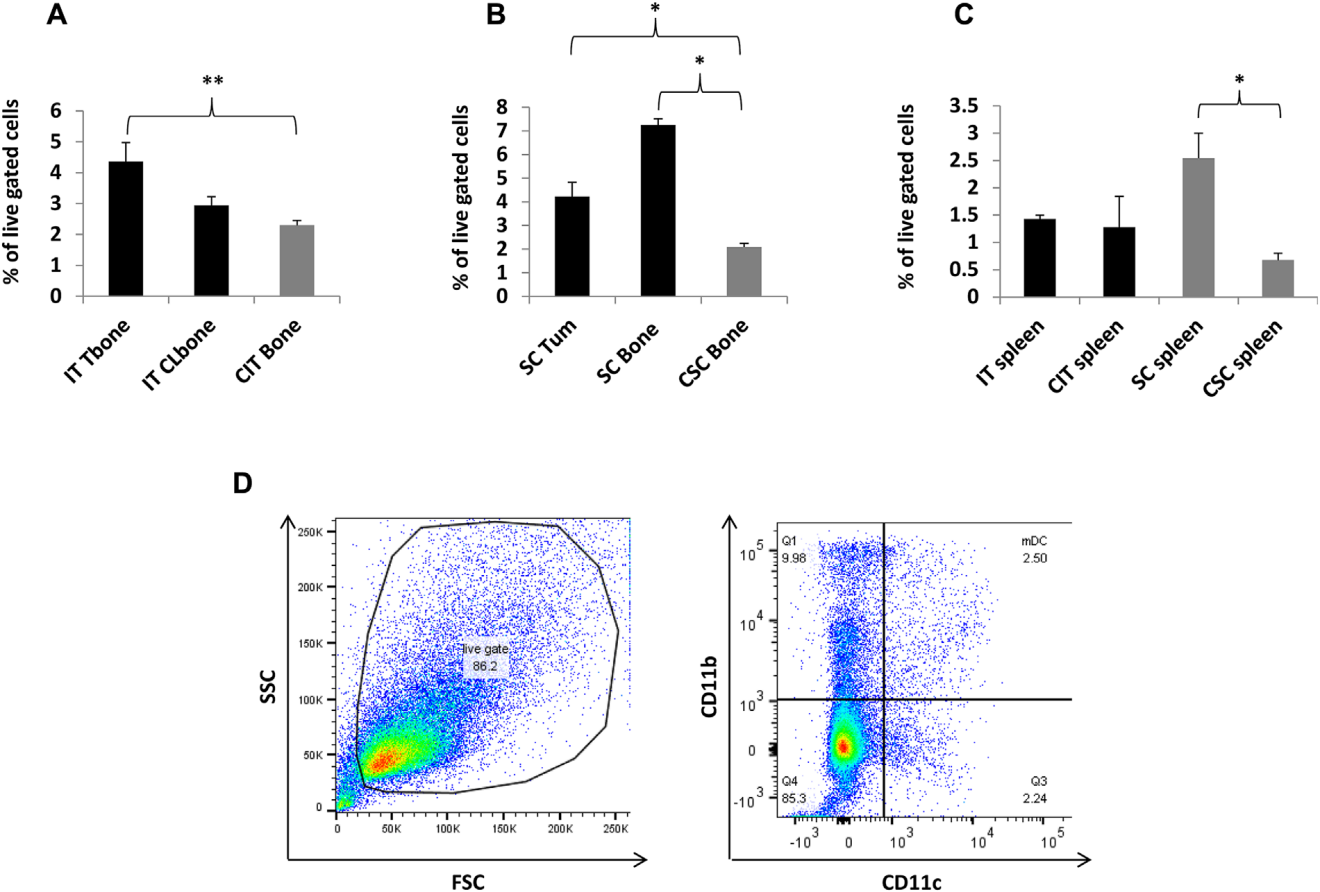
Comparative flow cytometry analysis of mDC in bone, spleen, and tumors: mDC were increased within the bone and subcutaneous TME's compared to controls (A and B, respectively). Whereas, intratibial tumor effects on mDC were confined to the TME, subcutaneous tumor effects on mDC extended beyond the TME with increases in non‐tumor bearing bone of mice with subcutaneous tumors compared to bone from control mice (B). The effect of subcutaneous tumor location was also evident in splenic tissue, while intratibial tumors played no significant role in altering splenic mDC populations (C). Representative flow cytometry gating for mDC is shown (D). Tissue sites examined were intratibial tumor injected bone (IT Tbone), intratibial contralateral bone (IT Clbone), PBS intratibial injected bone (CIT bone), spleen from intratibial tumor injected animals (IT spleen), spleen from intratibial PBS injected animals (CIT spleen), subcutaneous tumor (SC Tum), non‐tumor bearing bone from subcutaneous tumor injected mice (SC bone), bone from subcutaneous control mice (CSC bone), spleen from subcutaneous injected mice (SC spleen), spleen from subcutaneous control mice (CSC spleen) IT injected tumor group; *n* = 9, IT control group; *n* = 6, subcutaneous tumor group; *n* = 7, subcutaneous control group; *n* = 3. (*) denotes *p* < 0.05, (**) denotes *p* < 0.01.

Analysis of mDC revealed increases within intratibial and subcutaneous tumors. Whereas, the effects on mDC in intratibial tumors were confined to the TME, the effects on subcutaneous tumors extended beyond the TME. Both intratibial and subcutaneous tumors resulted in comparable mDC increases within the TME; 90% and 102% respective increases over controls (Fig. 3A and B, respectively). Meanwhile, only subcutaneous tumors resulted in an increase in mDC outside the TME. Bone from mice bearing only subcutaneous tumors had a 247% increase of mDC over bone of control mice (Fig. 3B). The increase in mDC following subcutaneous tumor growth also extended to the spleens by 275%, compared to controls (Fig. 3C). Representative flow cytometry gating for mDC is shown (Fig. 3D).

### Subcutaneous tumors affect T‐cell populations within, and beyond the TME

**Figure 4:**
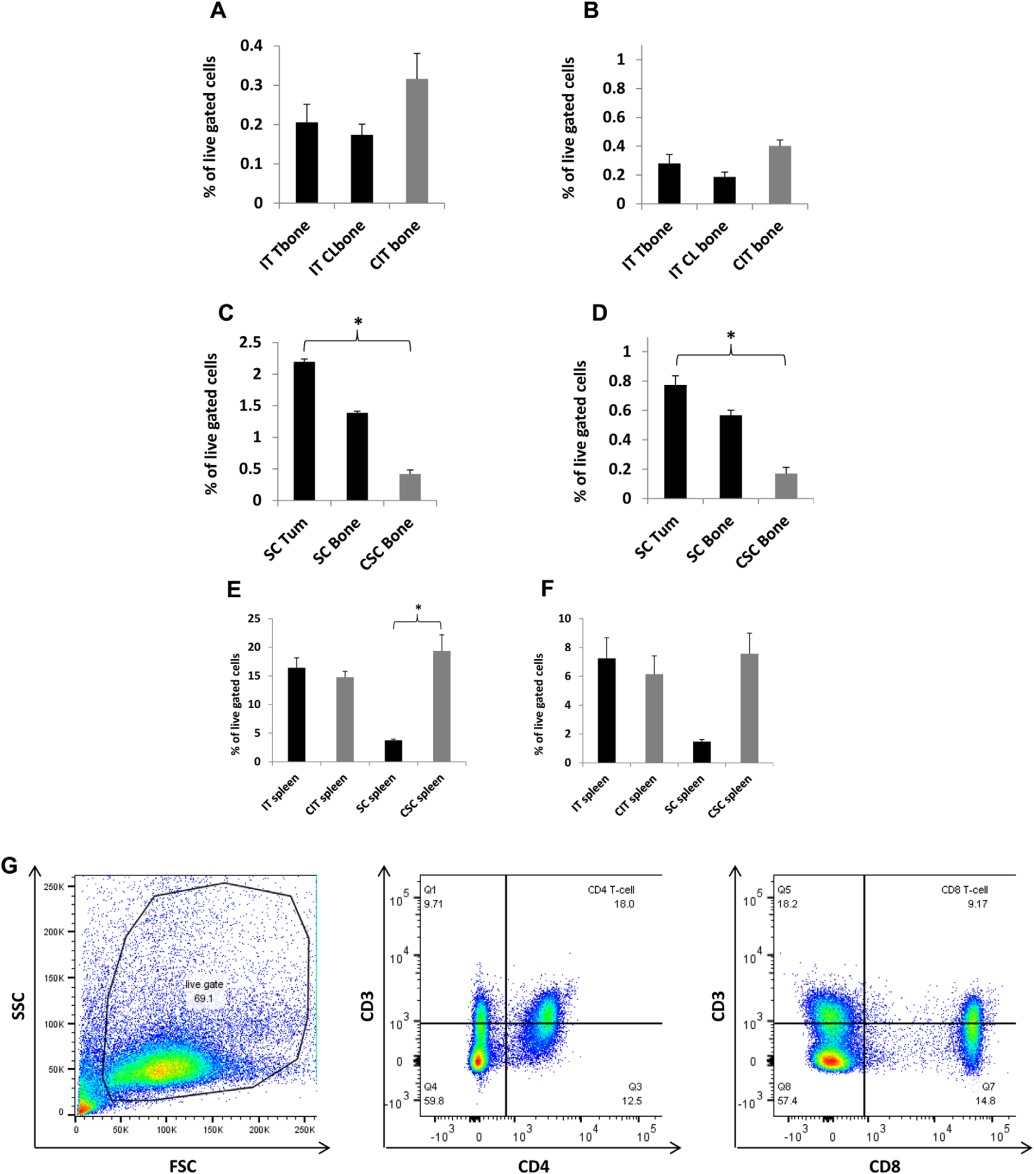
Comparative flow cytometry analysis of T‐cells in bone, spleen and tumors: Within the intratibial TME, CD4^+^ and CD8^+^ T‐cells had only minor decreases compared to PBS controls (A and B, respectively). The effect from subcutaneous tumors was profound (C and D). Tumors had significant increases and there was also an increase of CD4^+^ and CD8^+^ T‐cells in non‐tumor bearing bone of mice with subcutaneous tumors compared to controls (C and D). Spleens of intratibial tumor bearing mice had no significant differences for CD4^+^ and CD8^+^ T‐cells compared to controls (E and F, respectively). Spleens of subcutaneous tumor bearing mice had decreased CD4^+^ and CD8^+^ T‐cells compared to controls (E and F, respectively). Representative flow cytometry gating for CD4^+^ and CD8^+^ T‐cells is shown (4G). Tissue sites examined were intratibial tumor injected bone (IT Tbone), intratibial contralateral bone (IT Clbone), PBS intratibial injected bone (CIT bone), spleen from intratibial tumor injected animals (IT spleen), spleen from intratibial PBS injected animals (CIT spleen), subcutaneous tumor (SC Tum), non‐tumor bearing bone from subcutaneous tumor injected mice (SC bone), bone from subcutaneous control mice (CSC bone), spleen from subcutaneous injected mice (SC spleen), spleen from subcutaneous control mice (CSC spleen). IT injected tumor group; *n* = 9, IT control group; *n* = 6, subcutaneous tumor group; *n* = 7, subcutaneous control group; *n* = 3. (*) denotes *p* < 0.05.

T‐cells can both regulate, and be regulated by, macrophages and mDC 26, 27, 28, 29. The influence of T‐cells in regulating immune infiltrates within a tumor prompted us to examine if, and to what extent CD4^+^ and CD8^+^ T‐cells were affected by tumor location. CD4^+^ and CD8^+^ T‐cells within the intratibial TME remained largely unaffected with only slight decreases (Fig. 4A and B, respectively). Meanwhile, CD4^+^ and CD8^+^ T‐cells from the subcutaneous TME increased 422% and 355%, respectively over controls (*p* < 0.05; Fig. 4C and D, respectively). Additionally, bone from mice bearing only subcutaneous tumors had a 230% and 233% respective increase of CD4^+^ and CD8^+^ T‐cells compared to control mice bone (Fig. 4C and D, respectively). Spleens of intratibial tumor bearing mice had no significant differences in CD4^+^ and CD8^+^ T‐cells compared to spleens of control mice (Fig. 4E and F, respectively). Meanwhile, the spleens of subcutaneous tumor‐bearing mice had an 81% decrease in CD4^+^ and CD8^+^ T‐cells compared to controls (Fig. 4E and F, respectively). Representative flow cytometry gating for CD4^+^ and CD8^+^ T‐cells is shown (Fig. 4G).

Regulatory T‐cells (T‐regs) have been shown to increase with cancer and are a prominent contributor to cancer‐related immunosuppression; causing both inhibition of T‐cell proliferation and function 30. Therefore, we examined if some of the increased CD4^+^ T‐cells among the TME and tumor‐free bone of mice with subcutaneous tumors might be CD4^+^, CD25^+^, FoxP3^+^ T‐regs. Surprisingly, there were no differences at either site between tumor‐bearing or control mice (data not shown). Additionally, mice with intratibial tumors also had no significant differences in T‐regs compared to controls (data not shown).

## Discussion

Unraveling how tumors interactively regulate and conscript the immune system into exerting a global protumorigenic effect has become an important field of cancer research. A better understanding of this process promises to yield marked improvements in how we treat the disease. Cancer immunotherapy is becoming more prominent as a front‐line therapy and as an adjuvant to chemotherapeutic modalities 31, 32, 33. Therefore, understanding how locational differences effect tumor progression is vital when designing therapies based on immune modulation. For example, we have recently shown osteoclasts can be derived from MDSCs and their ability to undergo osteoclastogenesis was entirely dependent on being located within the bone tumor environment as opposed to MDSC in bone without tumor, spleen, or tumors elsewhere that do not undergo osteclastogenesis 22.

As location was determined to be critical for this process, we wanted to further study the role that tumor location might have on inducing alterations of other immune cell types, either in number or phenotype. Therefore, the purpose of this study was to ascertain if tumor location itself, effects changes to levels and/or phenotypes of various immune effectors, both within the local and global environments. For this, we examined if differences in immune effector cells occurred between tumors singularly localized within the bone microenvironment or a soft‐tissue site using transplantable, immunocompetent murine BCa models, and focused on three areas; the tumor microenvironment, non‐tumor bearing bone, and the spleen. Results of our study, using a BCa model supports previous study showing tumor location plays a significant role on intratumoral immune profiles that have potential to affect therapeutic response 6. Additionally, and for the first time to our knowledge, we also demonstrate that differential tumor locations affect immune infiltrates in other tissues differentially, implying a systemic effect dependent on tumor location. Our study demonstrated these affects occurred for macrophages, mDC and T‐cells.

As macrophage plasticity occurs between M1 versus M2 phenotypes, with the latter being protumorigenic and prominent within the TME, we examined if tumor location affects this phenotype. Surprisingly, we noted no differences, indicating that while tumor location altered macrophage numbers; it did not affect polarization. Since T‐regs numbers increase as a result of tumor progression, we looked at numbers of CD4^+^, CD25^+^, FoxP3^+^ T‐regs to see if the noted increases of CD4^+^ T‐cells resultant of subcutaneous tumors might be due to an increase in T‐regs. However, there was no difference for CD4^+^, CD25^+^, FoxP3^+^ T‐regs. Therefore, while tumor location affects T‐cell number, this increase was not attributable to T‐regs.

The noted differences in cell numbers would be expected to eventually result in differing cytokine profiles within the TME and globally, based on tumor location. Tumors stimulate pro‐tumorigenic cytokines production from mDC, macrophages, and T‐cells and the resident tissue type plays a pivotal role in this process 1, 3, 34. When macrophages entering the TME are subverted into a protumorigenic phenotype, they are often referred to as tumor associated macrophages (TAMs) and they display strong similarities to M2 phenotype macrophages 35. These TAMs are characterized by their secretion of the angiogenic factors; basic fibroblast growth factor, thymidine phosphorylase, urokinase‐type plasminogen activator, and adrenomedullin 36. They also secrete the immunosuppressive factors IL‐10 and TGF‐β, and IL‐10 from monocytes has been demonstrated to upregulate programmed cell death‐1 in an autocrine manner, leading to T‐cell dysfunction 37. Tumors subvert dendritic cell function and maturation through multiple mechanisms to create a protumorigenic environment. TGFβ and IL‐10 from tumors inhibit MHC class II and costimulatory molecules on dendritic cells, in addition to decreased IL‐12 production 38. Though we noted no differences in T‐regs; likely due to our early experimental time points, the higher levels of CD4^+^ T‐cells in both the TME and spleen of subcutaneous tumor bearing mice would likely result in greater T‐regs as compared to intratibial tumor bearing mice. T‐regs are immunosuppressive through multiple mechanisms. They suppress IL‐2 production by CD4^+^ and CD8^+^ T cells and inhibit cytotoxicity of CD8^+^ T cells 39. They inhibit CD80 and CD86 expression on antigen presenting cells, and stimulate their production of indoleamine 2,3‐dioxygenase, which catabolizes tryptophan, converting it to kynurenine, which kills T‐cells 40, 41. T‐regs are also capable of killing APCs, including B‐cells to further immunosuppression 42. Though we detected no phenotypical differences between cells of differing TME location; again due to our early time points examined in the study, it is apparent that each of the cells described can play pivotal protumorigenic roles via altered cytokine, ligand, and co‐stimulatory molecules profiles.

A summary of the cell types in given tissues and tumor location is shown with Table 1. Given that the noted cell types were affected to a greater extent in the subcutaneous TME versus the bone TME and also showed significance in non‐tumor bearing bone and spleens of mice with subcutaneous tumors, suggest that a solid tumor mass in soft tissue has a systemic regulation of immune effectors as compared to a tumor localized to bone. Though a detailed mechanism remains to be elucidated, our present finding that tumor location itself determines levels of immune infiltrates and whether this effect remains localized or disseminates systemically provides a clue on the role of TME in altering immune infiltrates. Utilizing renal, colon and prostate models, others have demonstrated similar effects that lend support to our findings 6. As metastatic disease locale can differ between patients, understanding the effect of tumor location on immune profile can provide translational insight into designing therapeutic strategies.

**Table 1:** Summary of immune cell characterizations by tumor locale

Cell type	Intratibial tumors	Subcutaneous tumors
MΦ		
Tumor	25% decrease	53% decrease
Non‐tumor bearing bone	n.s.	n.s.
Spleen	44% increase	214% increase
M1 versus M2	Followed Σ MΦ trends	Followed Σ MΦ trends
mDC		
Tumor	90% increase	102% increase
Non‐tumor bearing bone	n.s.	247% increase
Spleen	n.s.	275% increase
CD4^+^ and CD8^+^ T‐cells		
Tumor	n.s.	422% and 355% respective increase
Non‐tumor bearing bone	n.s.	230% and 233% respective increase
Spleen	n.s.	Both 81% decrease
T‐regs	n.s.	n.s.

## Author contributions

JAH and SP designed study strategy. JAH, VK, and SP designed experiments. JAH, VK, RA, CL performed experiments. GPS performed pathology assessments. JAH performed analysis. JAH prepared figures. JAH and SP prepared and revised the manuscript.

## Conflict of Interest

No financial or other conflicts exist with any of the authors, and the contents of this manuscript.

## Supporting information

Additional supporting information may be found in the online version of this article at the publisher's web‐site.
